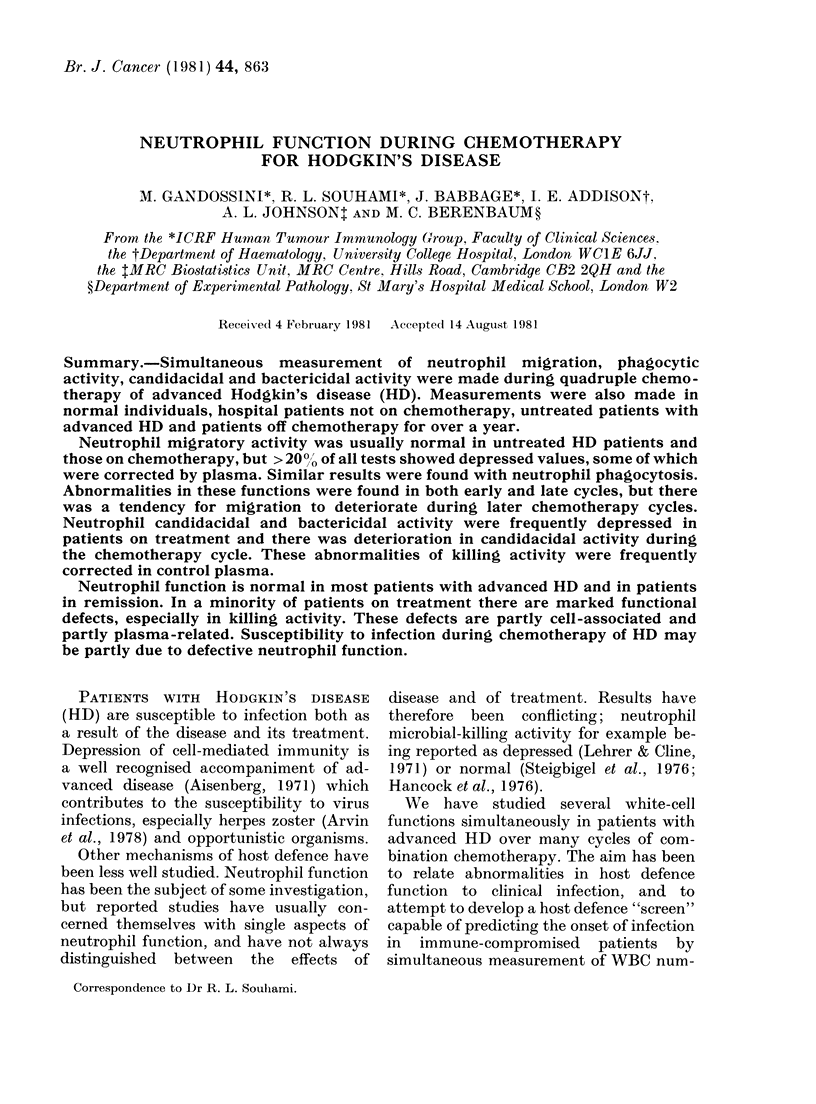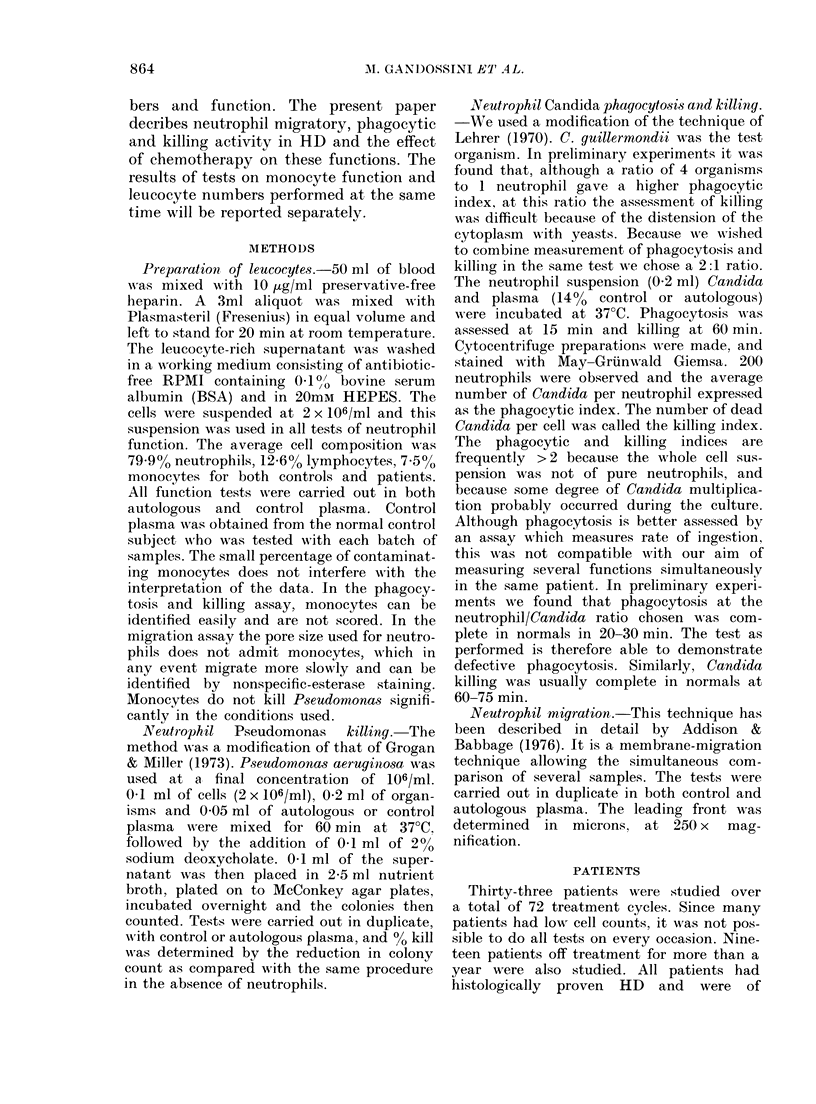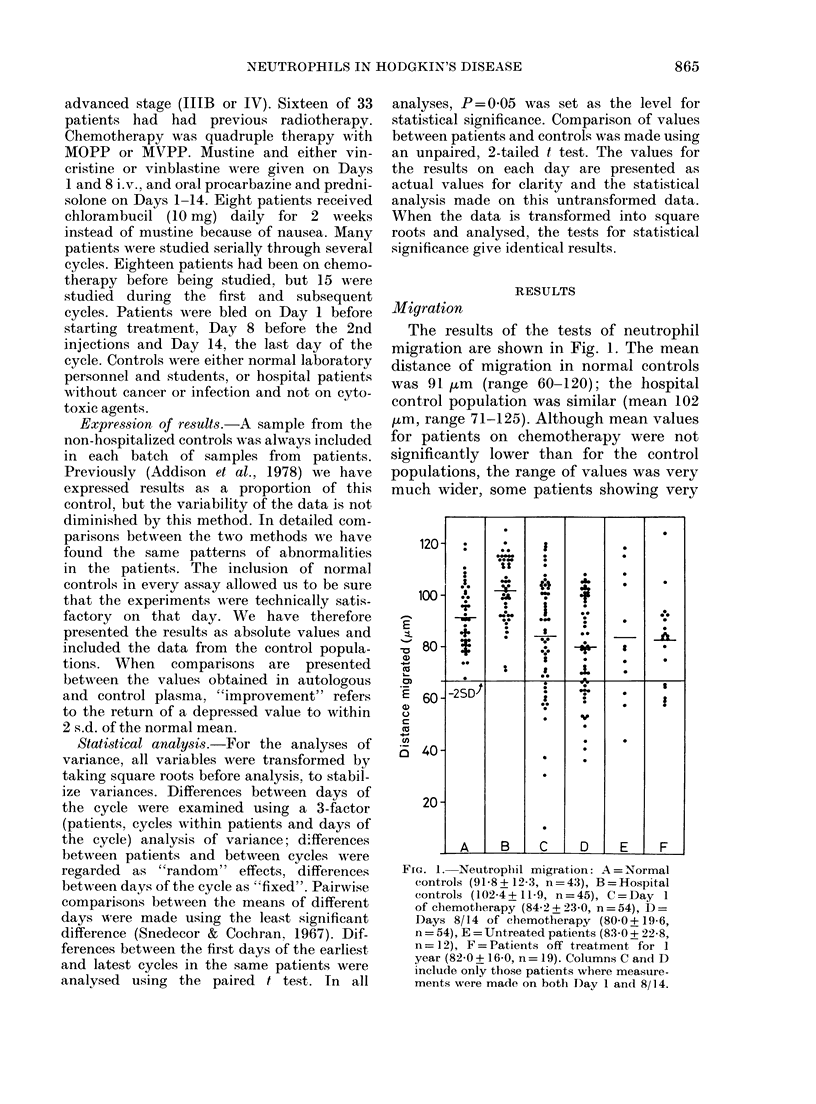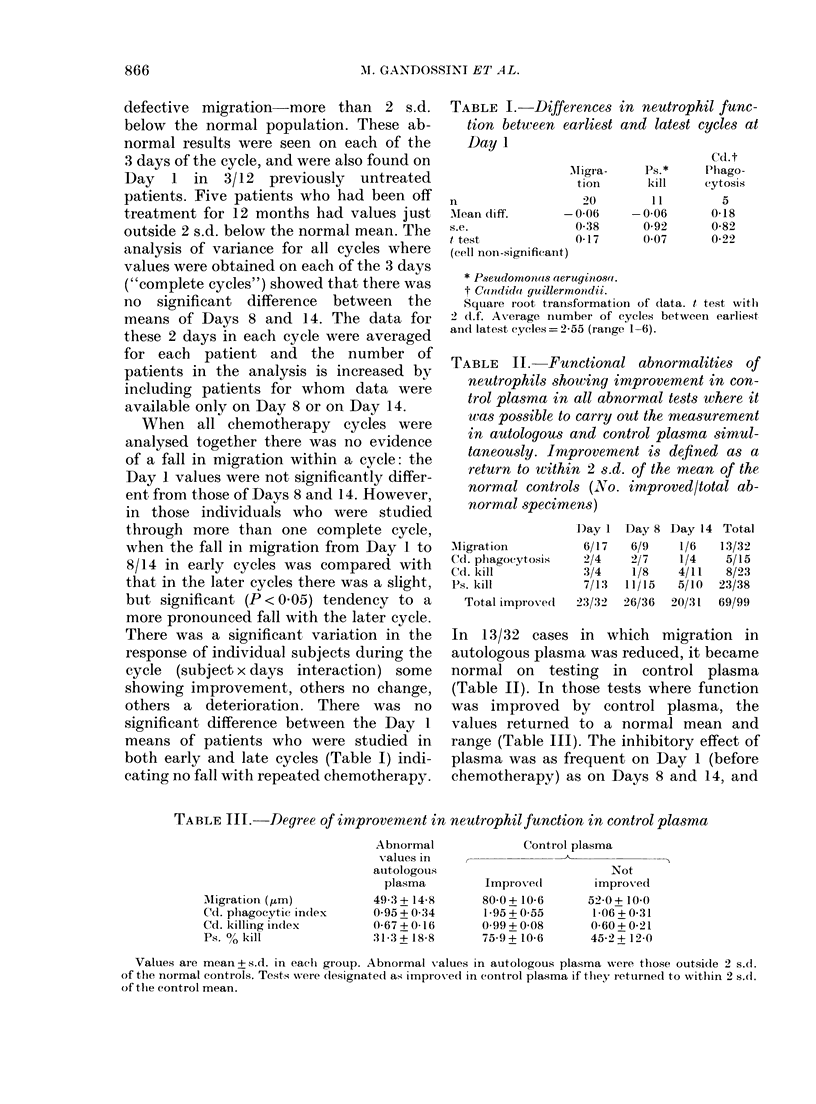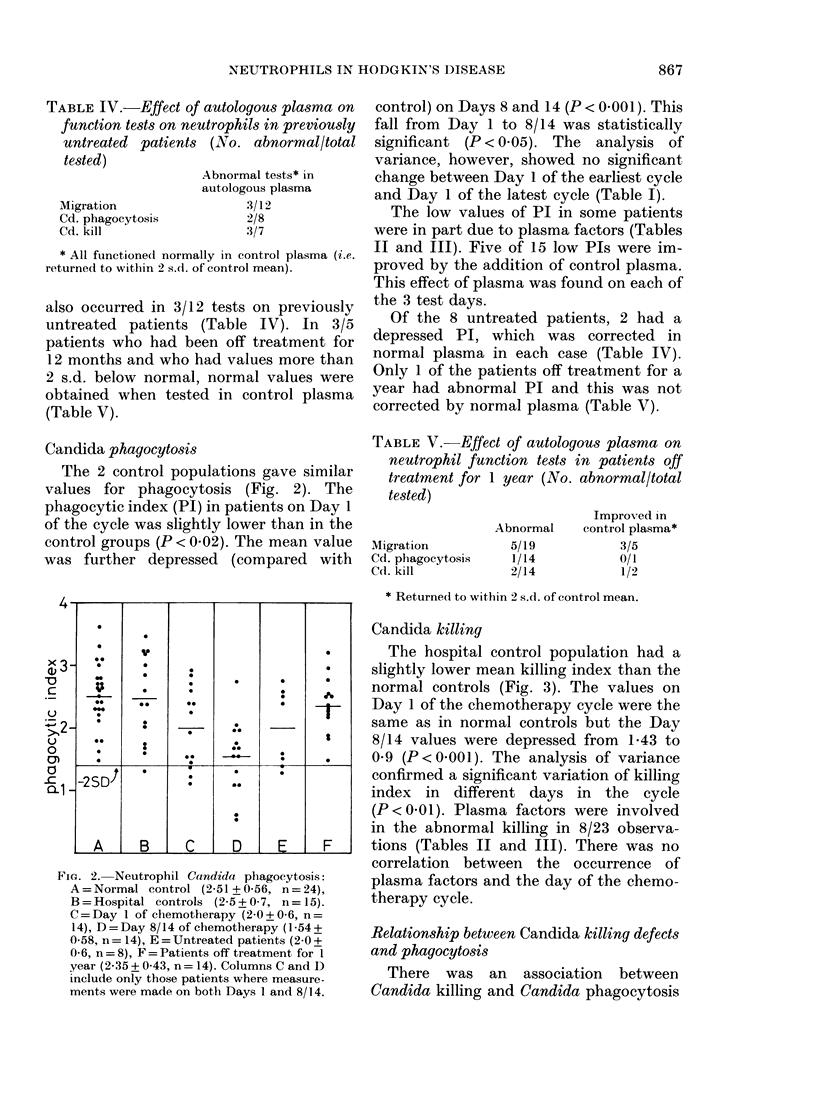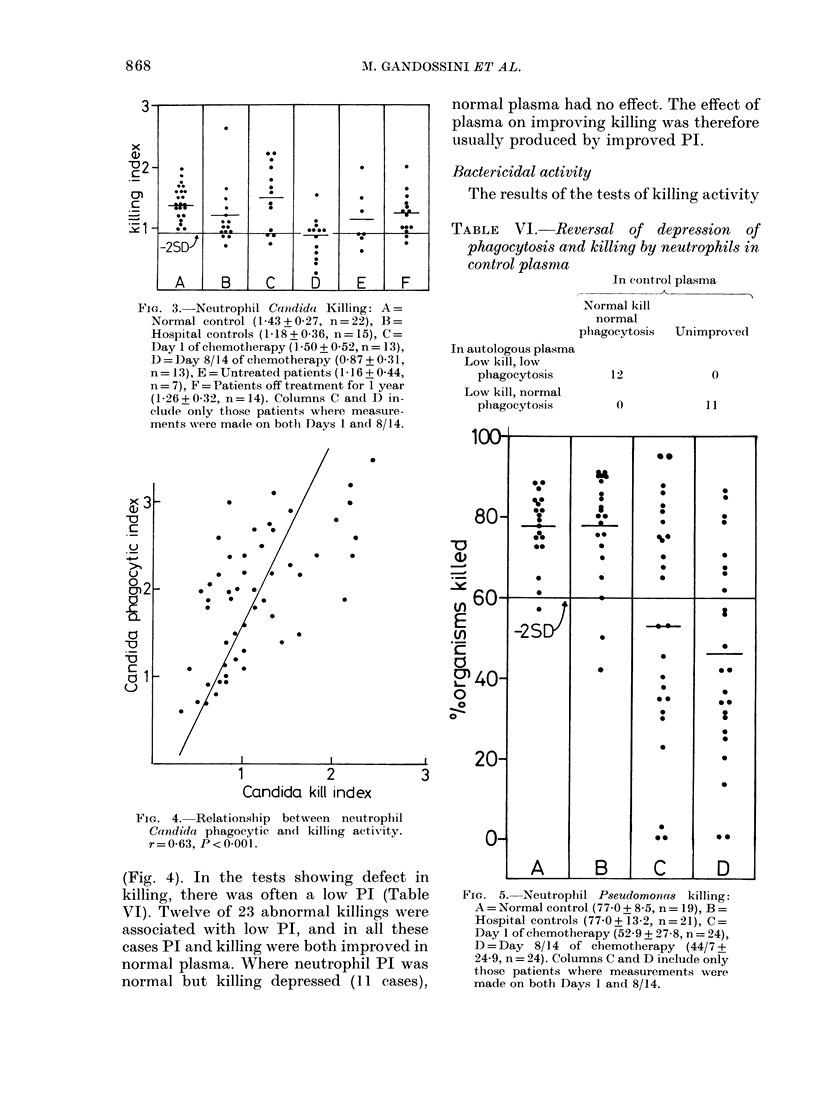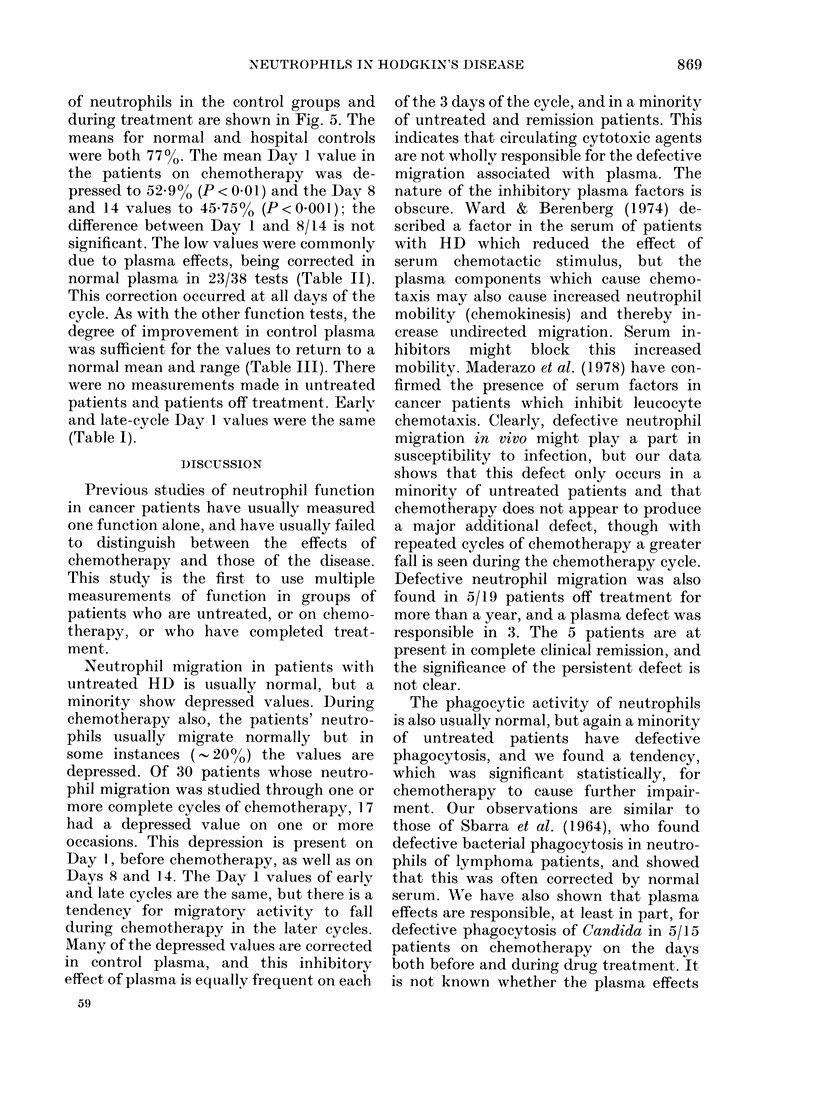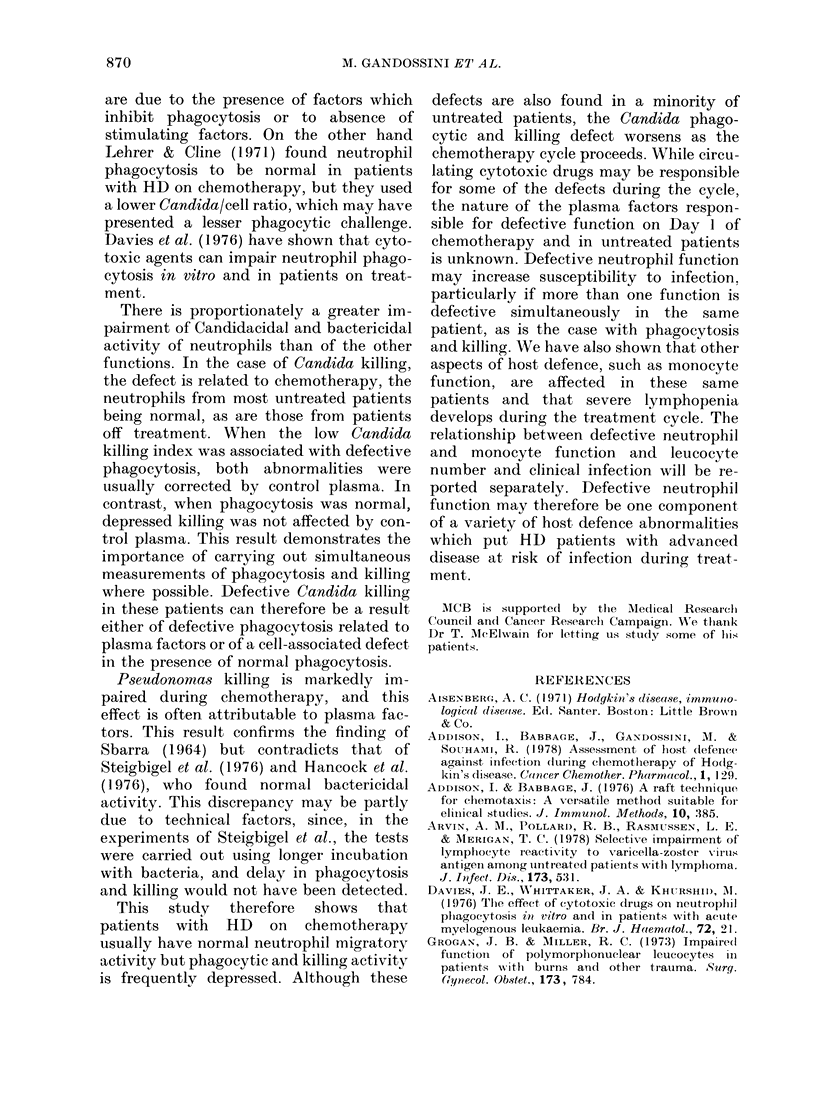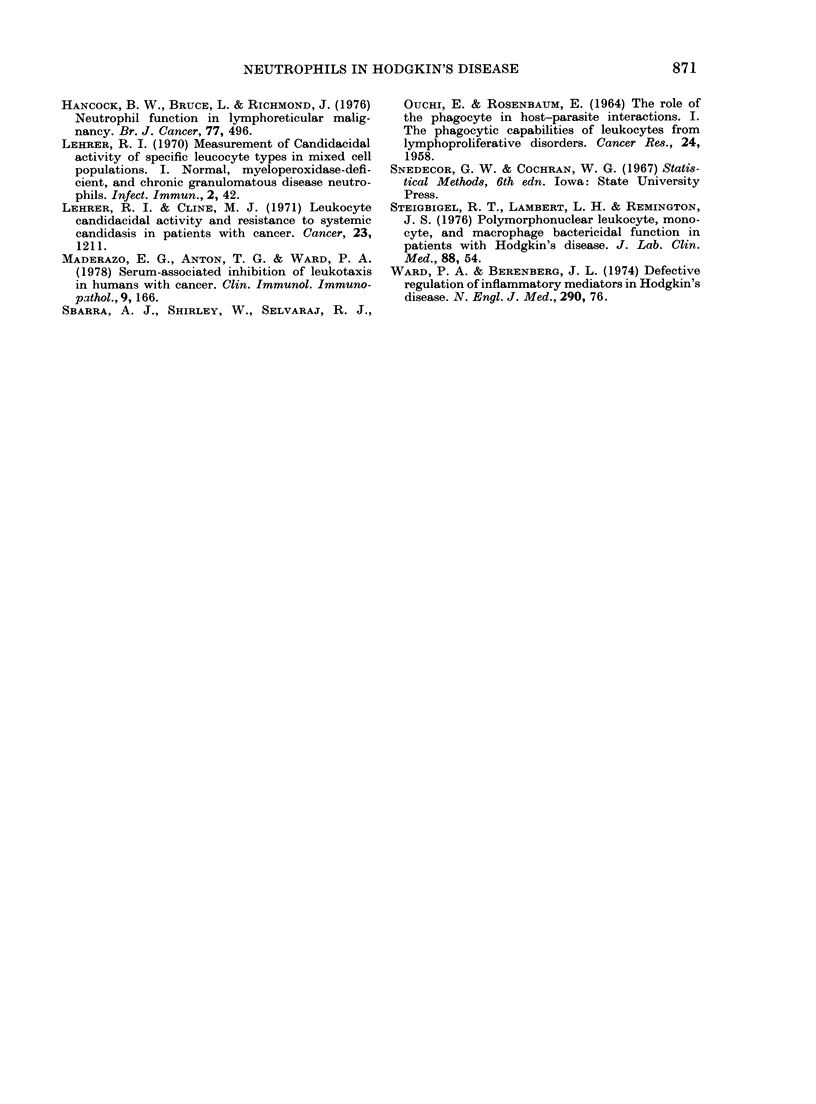# Neutrophil function during chemotherapy for Hodgkin's disease.

**DOI:** 10.1038/bjc.1981.285

**Published:** 1981-12

**Authors:** M. Gandossini, R. L. Souhami, J. Babbage, I. E. Addison, A. L. Johnson, M. C. Berenbaum

## Abstract

Simultaneous measurement of neutrophil migration, phagocytic activity, candidacidal and bactericidal activity were made during quadruple chemotherapy of advanced Hodgkin's disease (HD). Measurements were also made in normal individuals, hospital patients not on chemotherapy, untreated patients with advanced HD and patients off chemotherapy for over a year. Neutrophil migratory activity was usually normal in untreated HD patients and those on chemotherapy, but less than 20% of all tests showed depressed values, some of which were corrected by plasma. Similar results were found with neutrophil phagocytosis. Abnormalities in these functions were found in both early and late cycles, but there was a tendency for migration to deteriorate during later chemotherapy cycles. Neutrophil candidacidal and bactericidal activity were frequently depressed in patients on treatment and there was deterioration in candidacidal activity during the chemotherapy cycle. These abnormalities of killing activity were frequently corrected in control plasma. Neutrophil function is normal in most patients with advanced HD and in patients in remission. In a minority of patients on treatment there are marked functional defects, especially in killing activity. These defects are partly cell-associated and partly plasma-related. Susceptibility to infection during chemotherapy of HD may be partly due to defective neutrophil function.


*Br. J. Cancer* (1981) **44**, 863

### NEUTROPHIL FUNCTION DURING CHEMOTHERAPY FOR HODGKIN'S DISEASE

M. GANDOSSINI*, R. L. SOUHAMI*, J. BABBAGE*, I. E. ADDISON†, A. L. JOHNSON‡ AND M. C. BERENBAUM§

*From the *ICRF Human Tumour Immunology Group, Faculty of Clinical Sciences, the †Department of Haematology, University College Hospital, London WC1E 6JJ, the ‡MRC Biostatistics Unit, MRC Centre, Hills Road, Cambridge CB2 2QH and the §Department of Experimental Pathology, St Mary's Hospital Medical School, London W2*

Received 4 February 1981 Accepted 14 August 1981

**Summary.—Simultaneous measurement of neutrophil migration, phagocytic activity, candidacidal and bactericidal activity were made during quadruple chemotherapy of advanced Hodgkin's disease (HD). Measurements were also made in normal individuals, hospital patients not on chemotherapy, untreated patients with advanced HD and patients off chemotherapy for over a year.**

**Neutrophil migratory activity was usually normal in untreated HD patients and those on chemotherapy, but >20% of all tests showed depressed values, some of which were corrected by plasma. Similar results were found with neutrophil phagocytosis. Abnormalities in these functions were found in both early and late cycles, but there was a tendency for migration to deteriorate during later chemotherapy cycles. Neutrophil candidacidal and bactericidal activity were frequently depressed in patients on treatment and there was deterioration in candidacidal activity during the chemotherapy cycle. These abnormalities of killing activity were frequently corrected in control plasma.**

**Neutrophil function is normal in most patients with advanced HD and in patients in remission. In a minority of patients on treatment there are marked functional defects, especially in killing activity. These defects are partly cell-associated and partly plasma-related. Susceptibility to infection during chemotherapy of HD may be partly due to defective neutrophil function.**

PATIENTS WITH HODGKIN'S DISEASE (HD) are susceptible to infection both as a result of the disease and its treatment. Depression of cell-mediated immunity is a well recognised accompaniment of advanced disease (Aisenberg, 1971) which contributes to the susceptibility to virus infections, especially herpes zoster (Arvin *et al.*, 1978) and opportunistic organisms.

Other mechanisms of host defence have been less well studied. Neutrophil function has been the subject of some investigation, but reported studies have usually concerned themselves with single aspects of neutrophil function, and have not always distinguished between the effects of disease and of treatment. Results have therefore been conflicting; neutrophil microbial-killing activity for example being reported as depressed (Lehrer & Cline, 1971) or normal (Steigbigel *et al.*, 1976; Hancock *et al.*, 1976).

We have studied several white-cell functions simultaneously in patients with advanced HD over many cycles of combination chemotherapy. The aim has been to relate abnormalities in host defence function to clinical infection, and to attempt to develop a host defence "screen" capable of predicting the onset of infection in immune-compromised patients by simultaneous measurement of WBC num-

Correspondence to Dr R. L. Souhami.

bers and function. The present paper decribes neutrophil migratory, phagocytic and killing activity in HD and the effect of chemotherapy on these functions. The results of tests on monocyte function and leucocyte numbers performed at the same time will be reported separately.

### METHODS

*Preparation of leucocytes.*—50 ml of blood was mixed with 10 $\mu$g/ml preservative-free heparin. A 3ml aliquot was mixed with Plasmasteril (Fresenius) in equal volume and left to stand for 20 min at room temperature. The leucocyte-rich supernatant was washed in a working medium consisting of antibiotic-free RPMI containing 0·1% bovine serum albumin (BSA) and in 20mM HEPES. The cells were suspended at $2 \times 10^6$/ml and this suspension was used in all tests of neutrophil function. The average cell composition was 79·9% neutrophils, 12·6% lymphocytes, 7·5% monocytes for both controls and patients. All function tests were carried out in both autologous and control plasma. Control plasma was obtained from the normal control subject who was tested with each batch of samples. The small percentage of contaminating monocytes does not interfere with the interpretation of the data. In the phagocytosis and killing assay, monocytes can be identified easily and are not scored. In the migration assay the pore size used for neutrophils does not admit monocytes, which in any event migrate more slowly and can be identified by nonspecific-esterase staining. Monocytes do not kill *Pseudomonas* significantly in the conditions used.

*Neutrophil* Pseudomonas *killing.*—The method was a modification of that of Grogan & Miller (1973). *Pseudomonas aeruginosa* was used at a final concentration of $10^6$/ml. 0·1 ml of cells ($2 \times 10^6$/ml), 0·2 ml of organisms and 0·05 ml of autologous or control plasma were mixed for 60 min at 37°C, followed by the addition of 0·1 ml of 2% sodium deoxycholate. 0·1 ml of the supernatant was then placed in 2·5 ml nutrient broth, plated on to McConkey agar plates, incubated overnight and the colonies then counted. Tests were carried out in duplicate, with control or autologous plasma, and % kill was determined by the reduction in colony count as compared with the same procedure in the absence of neutrophils.

*Neutrophil* Candida *phagocytosis and killing.*—We used a modification of the technique of Lehrer (1970). *C. guillermondii* was the test organism. In preliminary experiments it was found that, although a ratio of 4 organisms to 1 neutrophil gave a higher phagocytic index, at this ratio the assessment of killing was difficult because of the distension of the cytoplasm with yeasts. Because we wished to combine measurement of phagocytosis and killing in the same test we chose a 2:1 ratio. The neutrophil suspension (0·2 ml) *Candida* and plasma (14% control or autologous) were incubated at 37°C. Phagocytosis was assessed at 15 min and killing at 60 min. Cytocentrifuge preparations were made, and stained with May–Grünwald Giemsa. 200 neutrophils were observed and the average number of *Candida* per neutrophil expressed as the phagocytic index. The number of dead *Candida* per cell was called the killing index. The phagocytic and killing indices are frequently $>2$ because the whole cell suspension was not of pure neutrophils, and because some degree of *Candida* multiplication probably occurred during the culture. Although phagocytosis is better assessed by an assay which measures rate of ingestion, this was not compatible with our aim of measuring several functions simultaneously in the same patient. In preliminary experiments we found that phagocytosis at the neutrophil/*Candida* ratio chosen was complete in normals in 20–30 min. The test as performed is therefore able to demonstrate defective phagocytosis. Similarly, *Candida* killing was usually complete in normals at 60–75 min.

*Neutrophil migration.*—This technique has been described in detail by Addison & Babbage (1976). It is a membrane-migration technique allowing the simultaneous comparison of several samples. The tests were carried out in duplicate in both control and autologous plasma. The leading front was determined in microns, at 250× magnification.

### PATIENTS

Thirty-three patients were studied over a total of 72 treatment cycles. Since many patients had low cell counts, it was not possible to do all tests on every occasion. Nineteen patients off treatment for more than a year were also studied. All patients had histologically proven HD and were of advanced stage (IIIB or IV). Sixteen of 33 patients had had previous radiotherapy. Chemotherapy was quadruple therapy with MOPP or MVPP. Mustine and either vincristine or vinblastine were given on Days 1 and 8 i.v., and oral procarbazine and prednisolone on Days 1–14. Eight patients received chlorambucil (10 mg) daily for 2 weeks instead of mustine because of nausea. Many patients were studied serially through several cycles. Eighteen patients had been on chemotherapy before being studied, but 15 were studied during the first and subsequent cycles. Patients were bled on Day 1 before starting treatment, Day 8 before the 2nd injections and Day 14, the last day of the cycle. Controls were either normal laboratory personnel and students, or hospital patients without cancer or infection and not on cytotoxic agents.

*Expression of results*.—A sample from the non-hospitalized controls was always included in each batch of samples from patients. Previously (Addison *et al*., 1978) we have expressed results as a proportion of this control, but the variability of the data is not diminished by this method. In detailed comparisons between the two methods we have found the same patterns of abnormalities in the patients. The inclusion of normal controls in every assay allowed us to be sure that the experiments were technically satisfactory on that day. We have therefore presented the results as absolute values and included the data from the control populations. When comparisons are presented between the values obtained in autologous and control plasma, "improvement" refers to the return of a depressed value to within 2 s.d. of the normal mean.

*Statistical analysis*.—For the analyses of variance, all variables were transformed by taking square roots before analysis, to stabilize variances. Differences between days of the cycle were examined using a 3-factor (patients, cycles within patients and days of the cycle) analysis of variance; differences between patients and between cycles were regarded as "random" effects, differences between days of the cycle as "fixed". Pairwise comparisons between the means of different days were made using the least significant difference (Snedecor & Cochran, 1967). Differences between the first days of the earliest and latest cycles in the same patients were analysed using the paired *t* test. In all analyses, $P = 0{\cdot}05$ was set as the level for statistical significance. Comparison of values between patients and controls was made using an unpaired, 2-tailed *t* test. The values for the results on each day are presented as actual values for clarity and the statistical analysis made on this untransformed data. When the data is transformed into square roots and analysed, the tests for statistical significance give identical results.

### RESULTS

### *Migration*

The results of the tests of neutrophil migration are shown in Fig. 1. The mean distance of migration in normal controls was 91 μm (range 60–120); the hospital control population was similar (mean 102 μm, range 71–125). Although mean values for patients on chemotherapy were not significantly lower than for the control populations, the range of values was very much wider, some patients showing very

FIG. 1.—Neutrophil migration: A = Normal controls ($91{\cdot}8 \pm 12{\cdot}3$, n = 43), B = Hospital controls ($102{\cdot}4 \pm 11{\cdot}9$, n = 45), C = Day 1 of chemotherapy ($84{\cdot}2 \pm 23{\cdot}0$, n = 54), D = Days 8/14 of chemotherapy ($80{\cdot}0 \pm 19{\cdot}6$, n = 54), E = Untreated patients ($83{\cdot}0 \pm 22{\cdot}8$, n = 12), F = Patients off treatment for 1 year ($82{\cdot}0 \pm 16{\cdot}0$, n = 19). Columns C and D include only those patients where measurements were made on both Day 1 and 8/14.

defective migration—more than 2 s.d. below the normal population. These abnormal results were seen on each of the 3 days of the cycle, and were also found on Day 1 in 3/12 previously untreated patients. Five patients who had been off treatment for 12 months had values just outside 2 s.d. below the normal mean. The analysis of variance for all cycles where values were obtained on each of the 3 days ("complete cycles") showed that there was no significant difference between the means of Days 8 and 14. The data for these 2 days in each cycle were averaged for each patient and the number of patients in the analysis is increased by including patients for whom data were available only on Day 8 or on Day 14.

When all chemotherapy cycles were analysed together there was no evidence of a fall in migration within a cycle: the Day 1 values were not significantly different from those of Days 8 and 14. However, in those individuals who were studied through more than one complete cycle, when the fall in migration from Day 1 to 8/14 in early cycles was compared with that in the later cycles there was a slight, but significant ($P < 0{\cdot}05$) tendency to a more pronounced fall with the later cycle. There was a significant variation in the response of individual subjects during the cycle (subject × days interaction) some showing improvement, others no change, others a deterioration. There was no significant difference between the Day 1 means of patients who were studied in both early and late cycles (Table I) indicating no fall with repeated chemotherapy.

TABLE I.—*Differences in neutrophil function between earliest and latest cycles at Day* 1

| | Migration | Ps.* kill | Cd.† Phagocytosis |
|---|---|---|---|
| n | 20 | 11 | 5 |
| Mean diff. | −0·06 | −0·06 | 0·18 |
| s.e. | 0·38 | 0·92 | 0·82 |
| *t* test | 0·17 | 0·07 | 0·22 |
| (cell non-significant) | | | |

* *Pseudomonas aeruginosa.*

† *Candida guillermondii.*

Square root transformation of data. *t* test with 2 d.f. Average number of cycles between earliest and latest cycles = 2·55 (range 1–6).

TABLE II.—*Functional abnormalities of neutrophils showing improvement in control plasma in all abnormal tests where it was possible to carry out the measurement in autologous and control plasma simultaneously. Improvement is defined as a return to within 2 s.d. of the mean of the normal controls (No. improved/total abnormal specimens)*

| | Day 1 | Day 8 | Day 14 | Total |
|---|---|---|---|---|
| Migration | 6/17 | 6/9 | 1/6 | 13/32 |
| Cd. phagocytosis | 2/4 | 2/7 | 1/4 | 5/15 |
| Cd. kill | 3/4 | 1/8 | 4/11 | 8/23 |
| Ps. kill | 7/13 | 11/15 | 5/10 | 23/38 |
| Total improved | 23/32 | 26/36 | 20/31 | 69/99 |

In 13/32 cases in which migration in autologous plasma was reduced, it became normal on testing in control plasma (Table II). In those tests where function was improved by control plasma, the values returned to a normal mean and range (Table III). The inhibitory effect of plasma was as frequent on Day 1 (before chemotherapy) as on Days 8 and 14, and

TABLE III.—*Degree of improvement in neutrophil function in control plasma*

| | Abnormal values in autologous plasma | Control plasma: Improved | Control plasma: Not improved |
|---|---|---|---|
| Migration ($\mu$m) | 49·3 ± 14·8 | 80·0 ± 10·6 | 52·0 ± 10·0 |
| Cd. phagocytic index | 0·95 ± 0·34 | 1·95 ± 0·55 | 1·06 ± 0·31 |
| Cd. killing index | 0·67 ± 0·16 | 0·99 ± 0·08 | 0·60 ± 0·21 |
| Ps. % kill | 31·3 ± 18·8 | 75·9 ± 10·6 | 45·2 ± 12·0 |

Values are mean ± s.d. in each group. Abnormal values in autologous plasma were those outside 2 s.d. of the normal controls. Tests were designated as improved in control plasma if they returned to within 2 s.d. of the control mean.

TABLE IV.—*Effect of autologous plasma on function tests on neutrophils in previously untreated patients (No. abnormal/total tested)*

| | Abnormal tests* in autologous plasma |
|---|---|
| Migration | 3/12 |
| Cd. phagocytosis | 2/8 |
| Cd. kill | 3/7 |

* All functioned normally in control plasma (*i.e.* returned to within 2 s.d. of control mean).

also occurred in 3/12 tests on previously untreated patients (Table IV). In 3/5 patients who had been off treatment for 12 months and who had values more than 2 s.d. below normal, normal values were obtained when tested in control plasma (Table V).

### Candida *phagocytosis*

The 2 control populations gave similar values for phagocytosis (Fig. 2). The phagocytic index (PI) in patients on Day 1 of the cycle was slightly lower than in the control groups ($P < 0{\cdot}02$). The mean value was further depressed (compared with control) on Days 8 and 14 ($P < 0{\cdot}001$). This fall from Day 1 to 8/14 was statistically significant ($P < 0{\cdot}05$). The analysis of variance, however, showed no significant change between Day 1 of the earliest cycle and Day 1 of the latest cycle (Table I).

The low values of PI in some patients were in part due to plasma factors (Tables II and III). Five of 15 low PIs were improved by the addition of control plasma. This effect of plasma was found on each of the 3 test days.

Of the 8 untreated patients, 2 had a depressed PI, which was corrected in normal plasma in each case (Table IV). Only 1 of the patients off treatment for a year had abnormal PI and this was not corrected by normal plasma (Table V).

FIG. 2.—Neutrophil *Candida* phagocytosis: A = Normal control (2·51 ± 0·56, n = 24), B = Hospital controls (2·5 ± 0·7, n = 15). C = Day 1 of chemotherapy (2·0 ± 0·6, n = 14), D = Day 8/14 of chemotherapy (1·54 ± 0·58, n = 14), E = Untreated patients (2·0 ± 0·6, n = 8), F = Patients off treatment for 1 year (2·35 ± 0·43, n = 14). Columns C and D include only those patients where measurements were made on both Days 1 and 8/14.

TABLE V.—*Effect of autologous plasma on neutrophil function tests in patients off treatment for 1 year (No. abnormal/total tested)*

| | Abnormal | Improved in control plasma* |
|---|---|---|
| Migration | 5/19 | 3/5 |
| Cd. phagocytosis | 1/14 | 0/1 |
| Cd. kill | 2/14 | 1/2 |

* Returned to within 2 s.d. of control mean.

### Candida *killing*

The hospital control population had a slightly lower mean killing index than the normal controls (Fig. 3). The values on Day 1 of the chemotherapy cycle were the same as in normal controls but the Day 8/14 values were depressed from 1·43 to 0·9 ($P < 0{\cdot}001$). The analysis of variance confirmed a significant variation of killing index in different days in the cycle ($P < 0{\cdot}01$). Plasma factors were involved in the abnormal killing in 8/23 observations (Tables II and III). There was no correlation between the occurrence of plasma factors and the day of the chemotherapy cycle.

### *Relationship between* Candida *killing defects and phagocytosis*

There was an association between *Candida* killing and *Candida* phagocytosis

FIG. 3.—Neutrophil *Candida* Killing: A = Normal control (1·43 ± 0·27, n = 22), B = Hospital controls (1·18 ± 0·36, n = 15), C = Day 1 of chemotherapy (1·50 ± 0·52, n = 13), D = Day 8/14 of chemotherapy (0·87 ± 0·31, n = 13), E = Untreated patients (1·16 ± 0·44, n = 7), F = Patients off treatment for 1 year (1·26 ± 0·32, n = 14). Columns C and D include only those patients where measurements were made on both Days 1 and 8/14.

FIG. 4.—Relationship between neutrophil *Candida* phagocytic and killing activity. $r = 0{\cdot}63$, $P < 0{\cdot}001$.

(Fig. 4). In the tests showing defect in killing, there was often a low PI (Table VI). Twelve of 23 abnormal killings were associated with low PI, and in all these cases PI and killing were both improved in normal plasma. Where neutrophil PI was normal but killing depressed (11 cases), normal plasma had no effect. The effect of plasma on improving killing was therefore usually produced by improved PI.

*Bactericidal activity*

The results of the tests of killing activity

TABLE VI.—*Reversal of depression of phagocytosis and killing by neutrophils in control plasma*

| | In control plasma | |
|---|---|---|
| | Normal kill normal phagocytosis | Unimproved |
| In autologous plasma | | |
| Low kill, low phagocytosis | 12 | 0 |
| Low kill, normal phagocytosis | 0 | 11 |

FIG. 5.—Neutrophil *Pseudomonas* killing: A = Normal control (77·0 ± 8·5, n = 19), B = Hospital controls (77·0 ± 13·2, n = 21), C = Day 1 of chemotherapy (52·9 ± 27·8, n = 24), D = Day 8/14 of chemotherapy (44/7 ± 24·9, n = 24). Columns C and D include only those patients where measurements were made on both Days 1 and 8/14.

of neutrophils in the control groups and during treatment are shown in Fig. 5. The means for normal and hospital controls were both 77%. The mean Day 1 value in the patients on chemotherapy was depressed to 52·9% ($P < 0{\cdot}01$) and the Day 8 and 14 values to 45·75% ($P < 0{\cdot}001$); the difference between Day 1 and 8/14 is not significant. The low values were commonly due to plasma effects, being corrected in normal plasma in 23/38 tests (Table II). This correction occurred at all days of the cycle. As with the other function tests, the degree of improvement in control plasma was sufficient for the values to return to a normal mean and range (Table III). There were no measurements made in untreated patients and patients off treatment. Early and late-cycle Day 1 values were the same (Table I).

### DISCUSSION

Previous studies of neutrophil function in cancer patients have usually measured one function alone, and have usually failed to distinguish between the effects of chemotherapy and those of the disease. This study is the first to use multiple measurements of function in groups of patients who are untreated, or on chemotherapy, or who have completed treatment.

Neutrophil migration in patients with untreated HD is usually normal, but a minority show depressed values. During chemotherapy also, the patients' neutrophils usually migrate normally but in some instances ($\sim 20\%$) the values are depressed. Of 30 patients whose neutrophil migration was studied through one or more complete cycles of chemotherapy, 17 had a depressed value on one or more occasions. This depression is present on Day 1, before chemotherapy, as well as on Days 8 and 14. The Day 1 values of early and late cycles are the same, but there is a tendency for migratory activity to fall during chemotherapy in the later cycles. Many of the depressed values are corrected in control plasma, and this inhibitory effect of plasma is equally frequent on each of the 3 days of the cycle, and in a minority of untreated and remission patients. This indicates that circulating cytotoxic agents are not wholly responsible for the defective migration associated with plasma. The nature of the inhibitory plasma factors is obscure. Ward & Berenberg (1974) described a factor in the serum of patients with HD which reduced the effect of serum chemotactic stimulus, but the plasma components which cause chemotaxis may also cause increased neutrophil mobility (chemokinesis) and thereby increase undirected migration. Serum inhibitors might block this increased mobility. Maderazo *et al.* (1978) have confirmed the presence of serum factors in cancer patients which inhibit leucocyte chemotaxis. Clearly, defective neutrophil migration *in vivo* might play a part in susceptibility to infection, but our data shows that this defect only occurs in a minority of untreated patients and that chemotherapy does not appear to produce a major additional defect, though with repeated cycles of chemotherapy a greater fall is seen during the chemotherapy cycle. Defective neutrophil migration was also found in 5/19 patients off treatment for more than a year, and a plasma defect was responsible in 3. The 5 patients are at present in complete clinical remission, and the significance of the persistent defect is not clear.

The phagocytic activity of neutrophils is also usually normal, but again a minority of untreated patients have defective phagocytosis, and we found a tendency, which was significant statistically, for chemotherapy to cause further impairment. Our observations are similar to those of Sbarra *et al.* (1964), who found defective bacterial phagocytosis in neutrophils of lymphoma patients, and showed that this was often corrected by normal serum. We have also shown that plasma effects are responsible, at least in part, for defective phagocytosis of *Candida* in 5/15 patients on chemotherapy on the days both before and during drug treatment. It is not known whether the plasma effects are due to the presence of factors which inhibit phagocytosis or to absence of stimulating factors. On the other hand Lehrer & Cline (1971) found neutrophil phagocytosis to be normal in patients with HD on chemotherapy, but they used a lower *Candida*/cell ratio, which may have presented a lesser phagocytic challenge. Davies *et al.* (1976) have shown that cytotoxic agents can impair neutrophil phagocytosis *in vitro* and in patients on treatment.

There is proportionately a greater impairment of Candidacidal and bactericidal activity of neutrophils than of the other functions. In the case of *Candida* killing, the defect is related to chemotherapy, the neutrophils from most untreated patients being normal, as are those from patients off treatment. When the low *Candida* killing index was associated with defective phagocytosis, both abnormalities were usually corrected by control plasma. In contrast, when phagocytosis was normal, depressed killing was not affected by control plasma. This result demonstrates the importance of carrying out simultaneous measurements of phagocytosis and killing where possible. Defective *Candida* killing in these patients can therefore be a result either of defective phagocytosis related to plasma factors or of a cell-associated defect in the presence of normal phagocytosis.

*Pseudonomas* killing is markedly impaired during chemotherapy, and this effect is often attributable to plasma factors. This result confirms the finding of Sbarra (1964) but contradicts that of Steigbigel *et al.* (1976) and Hancock *et al.* (1976), who found normal bactericidal activity. This discrepancy may be partly due to technical factors, since, in the experiments of Steigbigel *et al.*, the tests were carried out using longer incubation with bacteria, and delay in phagocytosis and killing would not have been detected.

This study therefore shows that patients with HD on chemotherapy usually have normal neutrophil migratory activity but phagocytic and killing activity is frequently depressed. Although these defects are also found in a minority of untreated patients, the *Candida* phagocytic and killing defect worsens as the chemotherapy cycle proceeds. While circulating cytotoxic drugs may be responsible for some of the defects during the cycle, the nature of the plasma factors responsible for defective function on Day 1 of chemotherapy and in untreated patients is unknown. Defective neutrophil function may increase susceptibility to infection, particularly if more than one function is defective simultaneously in the same patient, as is the case with phagocytosis and killing. We have also shown that other aspects of host defence, such as monocyte function, are affected in these same patients and that severe lymphopenia develops during the treatment cycle. The relationship between defective neutrophil and monocyte function and leucocyte number and clinical infection will be reported separately. Defective neutrophil function may therefore be one component of a variety of host defence abnormalities which put HD patients with advanced disease at risk of infection during treatment.

MCB is supported by the Medical Research Council and Cancer Research Campaign. We thank Dr T. McElwain for letting us study some of his patients.

### REFERENCES

AISENBERG, A. C. (1971) *Hodgkin's disease, immunological disease*. Ed. Santer. Boston: Little Brown & Co.

ADDISON, I., BABBAGE, J., GANDOSSINI, M. & SOUHAMI, R. (1978) Assessment of host defence against infection during chemotherapy of Hodgkin's disease. *Cancer Chemother. Pharmacol.*, **1**, 129.

ADDISON, I. & BABBAGE, J. (1976) A raft technique for chemotaxis: A versatile method suitable for clinical studies. *J. Immunol. Methods*, **10**, 385.

ARVIN, A. M., POLLARD, R. B., RASMUSSEN, L. E. & MERIGAN, T. C. (1978) Selective impairment of lymphocyte reactivity to varicella-zoster virus antigen among untreated patients with lymphoma. *J. Infect. Dis.*, **173**, 531.

DAVIES, J. E., WHITTAKER, J. A. & KHURSHID, M. (1976) The effect of cytotoxic drugs on neutrophil phagocytosis *in vitro* and in patients with acute myelogenous leukaemia. *Br. J. Haematol.*, **72**, 21.

GROGAN, J. B. & MILLER, R. C. (1973) Impaired function of polymorphonuclear leucocytes in patients with burns and other trauma. *Surg. Gynecol. Obstet.*, **173**, 784.

HANCOCK, B. W., BRUCE, L. & RICHMOND, J. (1976) Neutrophil function in lymphoreticular malignancy. *Br. J. Cancer*, **77**, 496.

LEHRER, R. I. (1970) Measurement of Candidacidal activity of specific leucocyte types in mixed cell populations. I. Normal, myeloperoxidase-deficient, and chronic granulomatous disease neutrophils. *Infect. Immun.*, **2**, 42.

LEHRER, R. I. & CLINE, M. J. (1971) Leukocyte candidacidal activity and resistance to systemic candidasis in patients with cancer. *Cancer*, **23**, 1211.

MADERAZO, E. G., ANTON, T. G. & WARD, P. A. (1978) Serum-associated inhibition of leukotaxis in humans with cancer. *Clin. Immunol. Immunopathol.*, **9**, 166.

SBARRA, A. J., SHIRLEY, W., SELVARAJ, R. J., OUCHI, E. & ROSENBAUM, E. (1964) The role of the phagocyte in host–parasite interactions. I. The phagocytic capabilities of leukocytes from lymphoproliferative disorders. *Cancer Res.*, **24**, 1958.

SNEDECOR, G. W. & COCHRAN, W. G. (1967) *Statistical Methods, 6th edn.* Iowa: State University Press.

STEIGBIGEL, R. T., LAMBERT, L. H. & REMINGTON, J. S. (1976) Polymorphonuclear leukocyte, monocyte, and macrophage bactericidal function in patients with Hodgkin's disease. *J. Lab. Clin. Med.*, **88**, 54.

WARD, P. A. & BERENBERG, J. L. (1974) Defective regulation of inflammatory mediators in Hodgkin's disease. *N. Engl. J. Med.*, **290**, 76.